# Supplementary material for: Amphiphilic nanocarrier-induced modulation of PLK1 and miR-34a leads to improved therapeutic response in pancreatic cancer
Source: Nat Commun. 2018 Jan 2;9:16. doi: 10.1038/s41467-017-02283-9 (PMC5750234; doi:10.1038/s41467-017-02283-9)
Supplement: Supplementary file 1 — Supplementary Information [file 41467_2017_2283_MOESM1_ESM.pdf]

		Sex		Age (Years)		Survival time (months)		PLK1*		MYC*		Bcl2*		MET*	
	Patient	LTS	STS	LTS	STS	LTS	STS	LTS	STS	LTS	STS	LTS	STS	LTS	STS
	1	F	F	68.4	75.2	36.5	3.4	1	2	0	2	0	0	2	2
	2	M	M	75.3	56.7	27.6	7.3	1	2	0	3	0	0	3	2
	3	M	F	62.6	51.8	25.2	2.9	1	3	0	3	2	0	3	3
	4	M		70.0		20.9		2		0		1		3	
	5	M		49.9		25.2		1		2		0		2	
	6	F		74.1		8.9		2		3		0		3	
	7	M		71.1		22.1		1		0		1		2	
Average				67.36	61.24	23.76	4.53	1.29	2.33	0.71	2.67	0.57	0.00	2.57	2.33
SEM				3.31	7.13	3.13	1.38	0.18	0.33	0.47	0.33	0.30	0.00	0.20	0.33
p value				0.39		0.01		0.02		0.04		0.26		0.54	

Supplementary Table 1. PDAC patient data. * = Histology score (0-3: 0- none, 1- weak, 2- moderate, 3- high) for PLK1, MYC, Bcl2 and MET immunostaining in FFPE specimens of LTS and STS PDAC patients based on pathologist blinded assessment. LTS = Long-term survival; STS = Short-term survival.

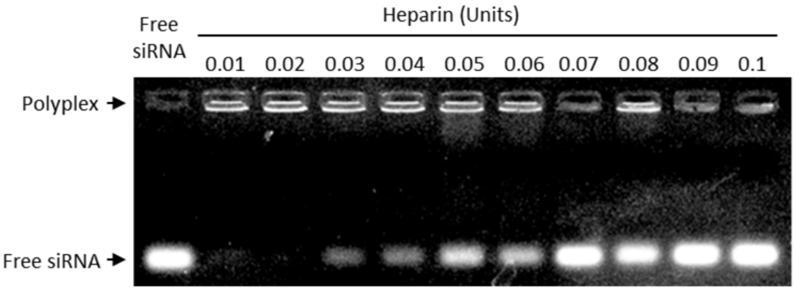

Supplementary Figure 1. PLK1-siRNA release from the polyplex obtained *in vitro* by the polyanion heparin displacement assay. Polyplexes of APA and PLK1-siRNA (N/P 2, 50 pmol siRNA) were incubated with increasing amount of heparin (0.01–0.1 IU) and run on 2% agarose gel (30 mim, 100 V).

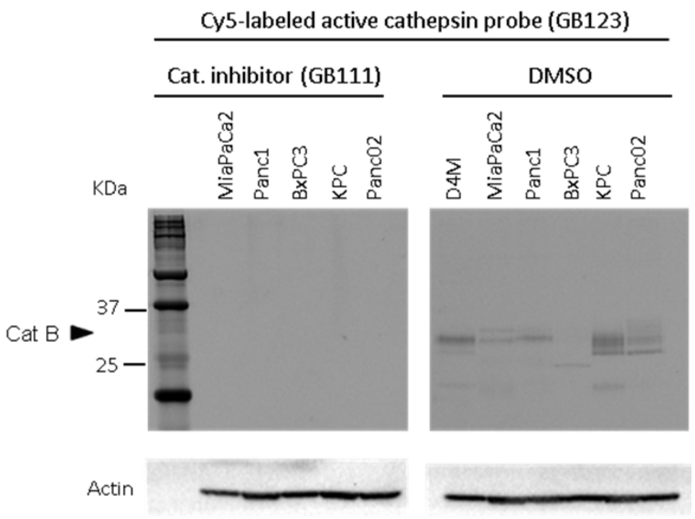

Supplementary Figure 2. Pancreatic cancer cell lines express cathepsin B. Expression level of cathepsins was evaluated using labeling of active cathepsins in intact PDAC cells. Human (MiaPaCa2, Panc1 and BxPC3) and murine (KPC and Panc02) PDAC cells (300,000 well^{-1}, 6-well plate) were seeded and 24 h later were pretreated with either 5 μM cathepsin inhibitor (GB111, not fluorescently labeled) or DMSO (0.1%). Then, all cells were incubated for 4 h with 2 μM Cy5-labeled activity-based probe GB123 (0.1% DMSO final concentration). To visualize active cathepsin labeling, treated cells were lysed, separated on SDS-PAGE and scanned by Typhoon scanner at excitation/emission filter set of 635/670 nm. After scanning, the gel was immunoblotted with actin antibody which served as loading control. The murine melanoma cell line D4M served as positive control for cathepsin labeling. Cat, Cathepsin.

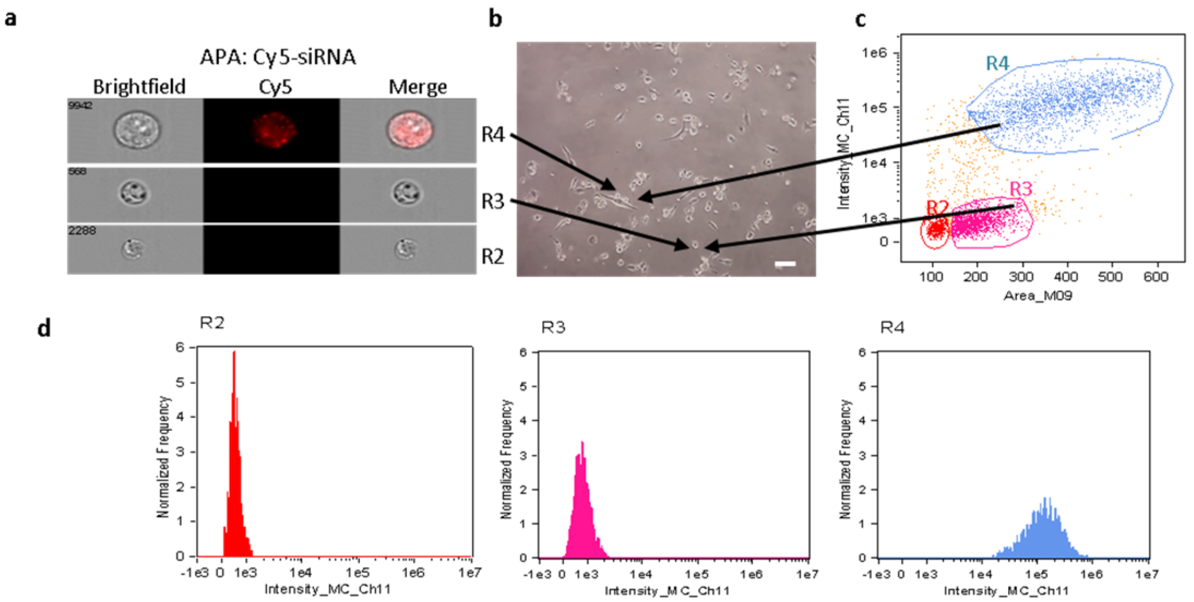

Supplementary Figure 3. Intracellular uptake is contributed by the adherent large cells population of MiaPaCa2. Internalization of Cy5-labeled siRNA into live MiaPaCa2 cells was followed using Imaging Flow Cytometer (ImageStream, Amnis). Live MiaPaCa2 cells (2 x 10^6 in 50 µl) were monitored 24 h following treatment with Cy5-labeled siRNA complexed with APA nanocarrier. **a**. Brightfield and fluorescent images of the cells according to their size R2, R3 and R4. **b**. Image of the cells acquired using a Nikon camera in magnification x100. Scale bar, 100 µm. **c**. Histogram that plots size (x axis) versus Cy5 intensity (y axis) showing the different cells populations. **d**. Internalization histograms of the different cells populations that plot the intensity of Cy5 (x axis) versus frequency (y axis).

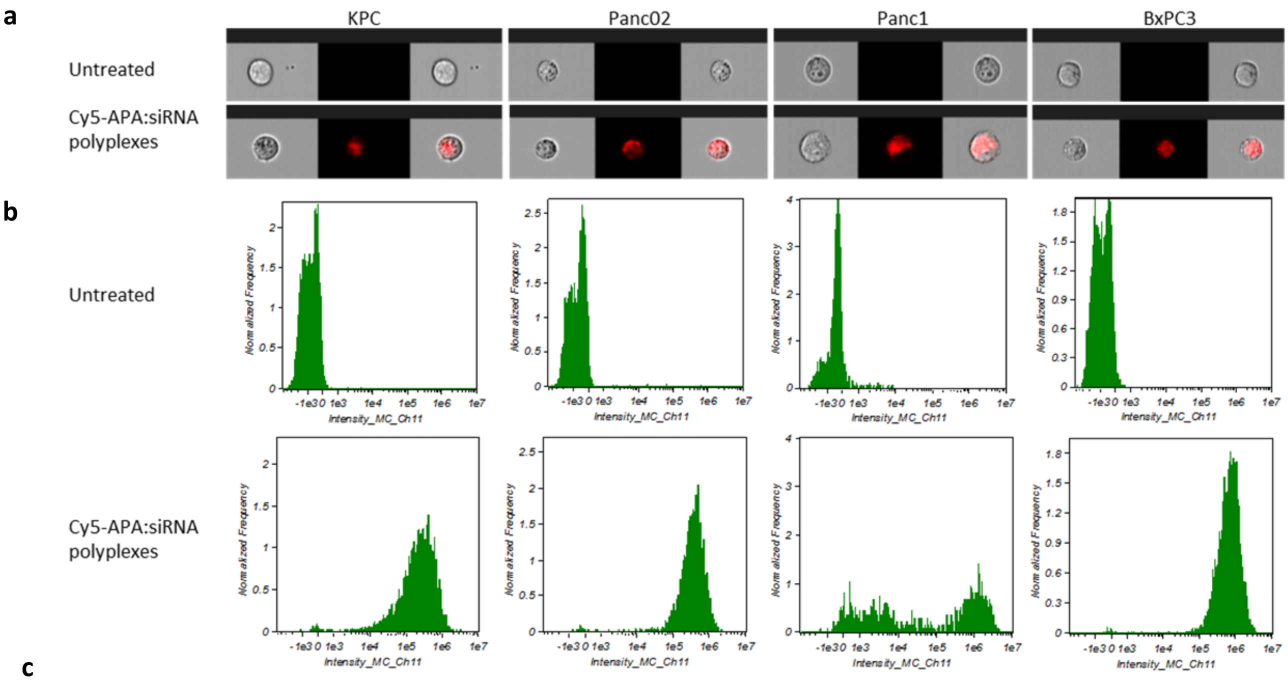

c

Tratment	Population	Count	%Gated	Internalization_ Erode, Median	Count	%Gated	Internalization_ Erode, Median	Count	%Gated	Internalization_ Erode, Median	Count	%Gated	Internalization_ Erode, Median
Untreated	singlet	4986	99.72	1.131	4994	99.88	1.584	1978	98.9	0.8264	4990	99.8	1.192
	pos	0	0	NaN	0	0	NaN	0	0	NaN	0	0	NaN
Cy5-PGAamine-siRNA polyplex	singlet	4993	99.86		4990	99.8		1343	99.04		4987	99.74	
	pos	4326	86.64	4.472	4862	97.43	3.073	628	46.76	2.729	4677	93.78	4.688

Supplementary Figure 4. Cellular internalization of nano-polyplexes into pancreatic cancer cell lines. KPC, Panc02, Panc1 and BxPC3 pancreatic cancer cells were incubated for 24 h with polyplexes containing Cy5-labeled APA (3.5 μg ml^{-1}) and siRNA (100 nM). Live cells (2 x 10^6 in 50 μl) were collected, washed with PBS and subjected to Imaging Flow Cytometer (ImageStream, Amnis). All treated cells were compared to untreated cells. Brightfield and fluorescent images of the cells (**a**), internalization histograms (**b**) and population statistics (**c**) are depicted.

Treatment	Cell viability (% of control)	Cell viability /100
miR-34a (100 nM)	66	0.66
PLK1-siRNA (50 nM)	70	0.70
miR+siRNA combined- expected	0.7*0.66	0.46
miR+siRNA combined- observed	24	0.24
Observed/Expected		0.52
0.52 < 0.8 → synergistic effect		

Supplementary Table 2. miR-34a and PLK1-siRNA effect on MiaPaCa2 cells viability is synergistic. The nature of the interaction between miR-34a and PLK1-siRNA was analyzed using the additive model. According to this model, a ratio between the observed and the expected viability of the cells was calculated for the combination treatment and a ratio less than 0.8 was considered to be synergistic.

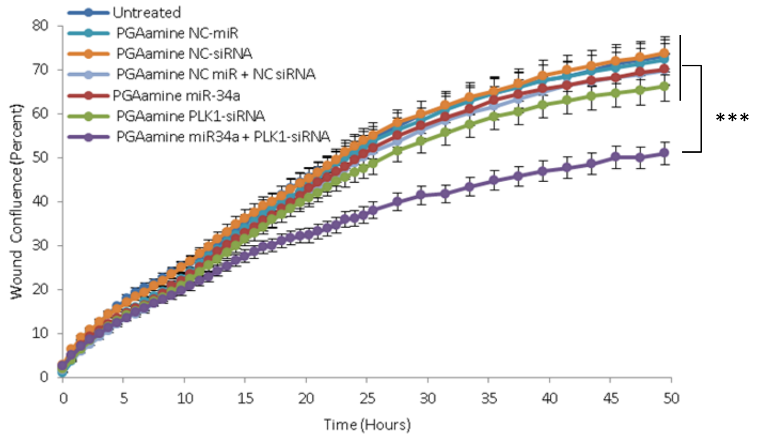

Supplementary Figure 5. MiaPaCa2 cells migration following treatment with polyplexes containing miRNA-siRNA. Wound confluence (percent out of initial wound at time 0) using IncuCyte live cell analysis system following treatments with APA polyplexes containing either miR-34a/NC-miR (100 nM) or PLK1-siRNA/NC-siRNA (50 nM) alone or their combinations. Data represent mean ± SD. (Student's *t*-test, ****P* < 0.001).

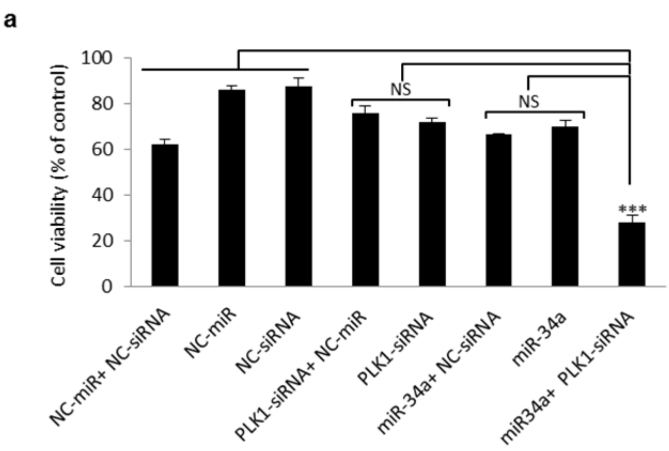

b

Treatment	Cell viability (% of control)	Cell viability /100
miR-34a (100 nM)	70	0.70
PLK1-siRNA (50 nM)	72	0.72
miR+siRNA combined-expected (0.7*0.72)		0.50
miR+siRNA combined-observed	28	0.28
Observed/Expected		0.56
0.56 < 0.8 → synergistic effect		

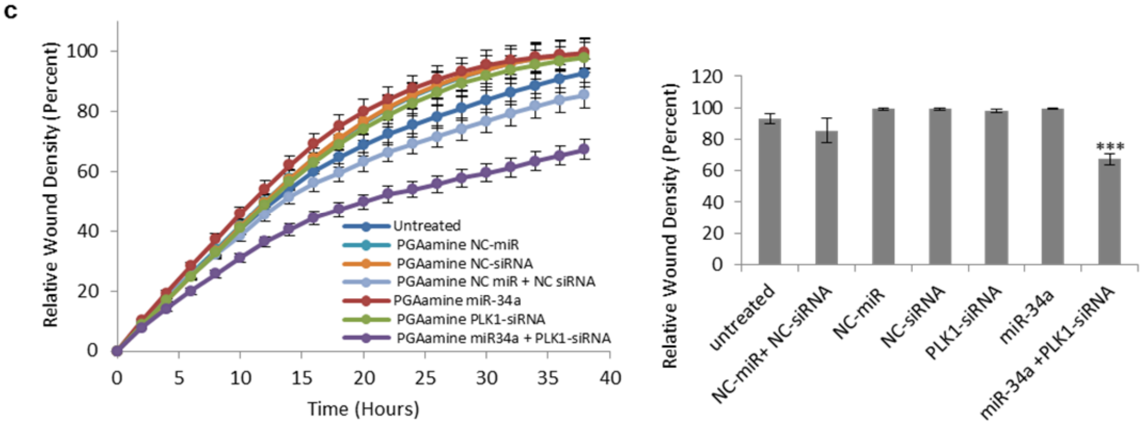

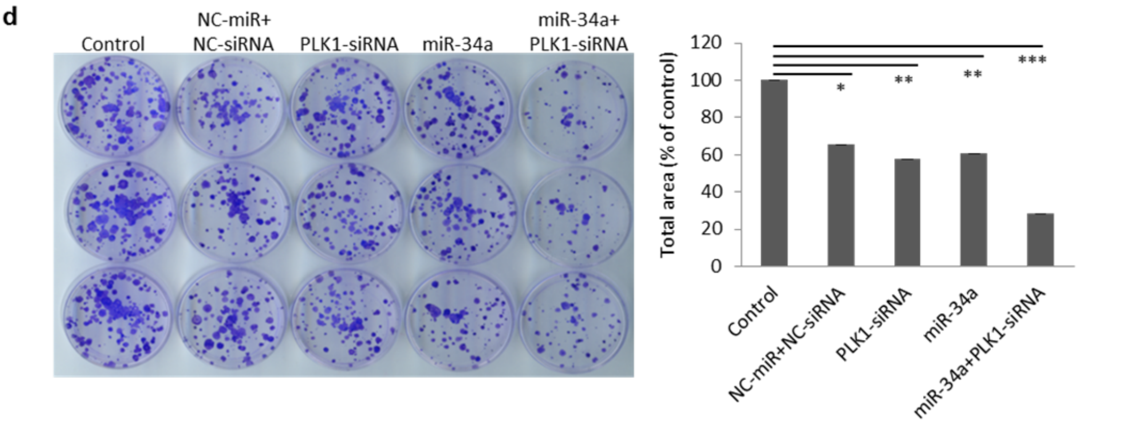

Supplementary Figure 6. The combination miR-34a and PLK1-siRNA inhibits KPC cells' viability, migration and survival. a. Proliferation following treatment with APA polyplexes containing miR-34a (100 nM) or PLK1-siRNA (50 nM) monotherapies or their combination **b**. A table summarizing calculations for the synergistic effect of the combined treatment according to the additive model. According to this model, a ratio between the observed and the expected viability of the cells was calculated for the combination treatment and a ratio less than 0.8 was considered as synergistic. **c**. Cells migration during 38 h following the same treatments as in (a), shown as relative wound density (percent out of initial wound at time 0) over time (left) and at end point of 38 h (right). **d**. Cell survival via colony formation assay for 8 days. A representative image of the colonies out of 3 independent biological repeats (Left) quantified for total area using ImageJ (right). Data represent mean ± SD. (Student's *t*-test, NS, not significant for $P > 0.05$, $*P < 0.05$, $**P < 0.01$, $***P < 0.001$).

a

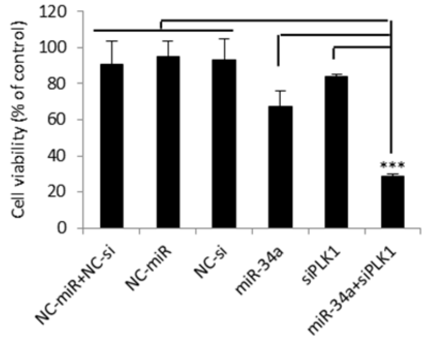

b

Traetment	Cell viability (% of control)	Cell viability/100
miR-34a (100 nM)	68	0.68
siPLK1 (50 nM)	84	0.84
miR+siRNA expected	0.68*0.84	0.57
miR+siRNA observed	29	0.29
Observed/Expected		0.51
0.51 <0.8 → synergistic effect		

c

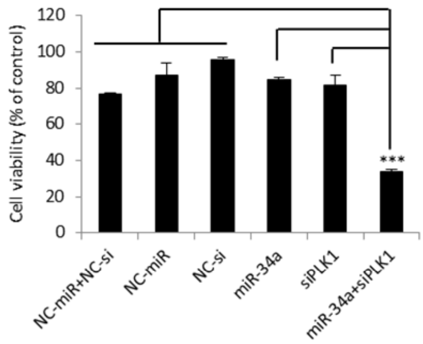

d

Traetment	Cell viability (% of control)	Cell viability/100
miR-34a (100 nM)	85	0.85
siPLK1 (50 nM)	82	0.82
miR+siRNA expected	0.81*0.94	0.70
miR+siRNA observed	34	0.34
Observed/Expected		0.49
0.49 <0.8 → synergistic effect		

e

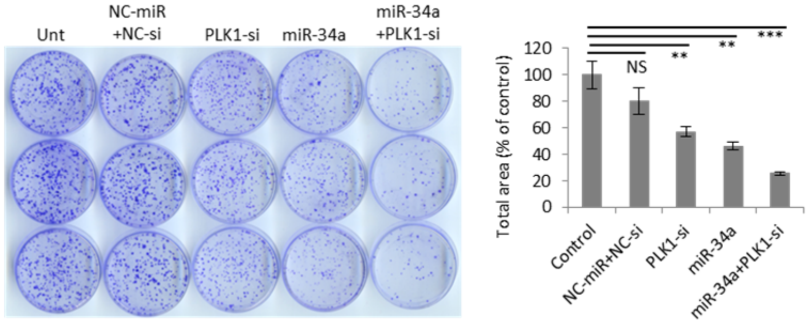

f

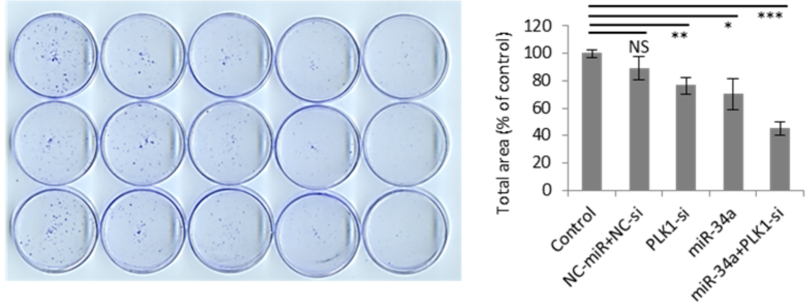

Supplementary Figure 7. The combination miR-34a and PLK1-siRNA inhibits viability and survival of human PDAC cells Panc1 and BxPC3. a. Panc1 cell viability following treatment with APA polyplexes containing miR-34a (100 nM) or PLK1-siRNA (50 nM) monotherapies or their combination. **b**. Synergistic effect of the combined treatment in Panc1 calculated according to the additive model. **c**. BxPC3 cell viability following the same treatments as in (a). **d**. Synergistic effect of the combined treatment in BxPC3. **e**, **f**. Cell survival of Panc1 (**e**) and BxPC3 (**f**) via colony formation assay for 14 days. Images of the colonies (Left) quantified for total area using ImageJ (right). All experiments included 3 biological repeats and were done in triplicates. All data represent mean ± SD. (Student's *t*-test, NS, not significant for $P > 0.05$, *$P < 0.05$, **$P < 0.01$, ***$P < 0.001$).

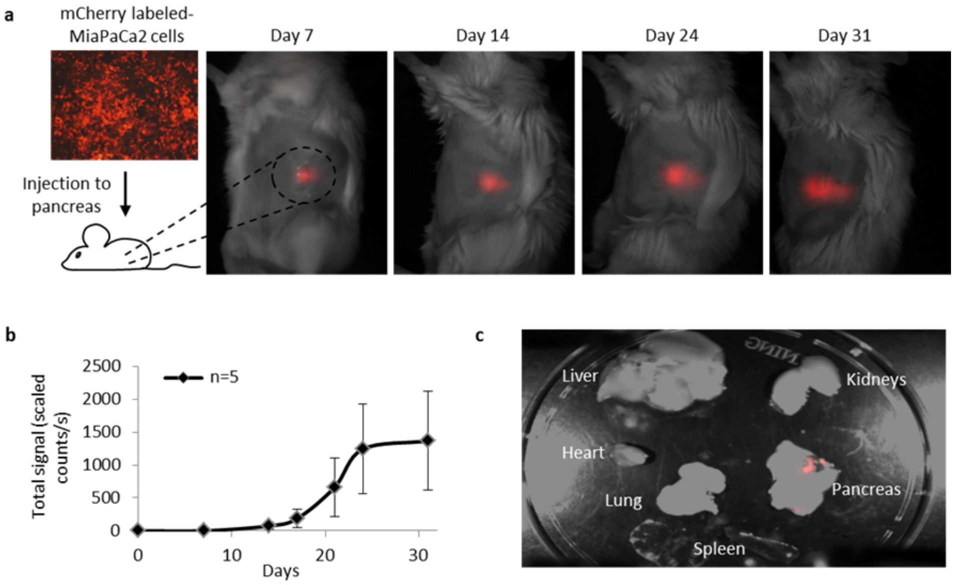

Supplementary Figure 8. Orthotopic PDAC mouse model of mCherry-labeled MiaPaCa2 cells. SCID male mice aged 5 weeks were anesthetized and $1x10^6$ mCherry-labeled MiaPaCa2 cells were injected orthotopically to the pancreas. Tumor growth was monitored by non-invasive intravital fluorescent microscopy. **a**. Imaging of pancreatic tumor in a live mouse. **b**. Pancreatic tumors growth rate. **c**. mCherry-fluorescent signal was found only in the pancreas. Data represent mean ± SEM.

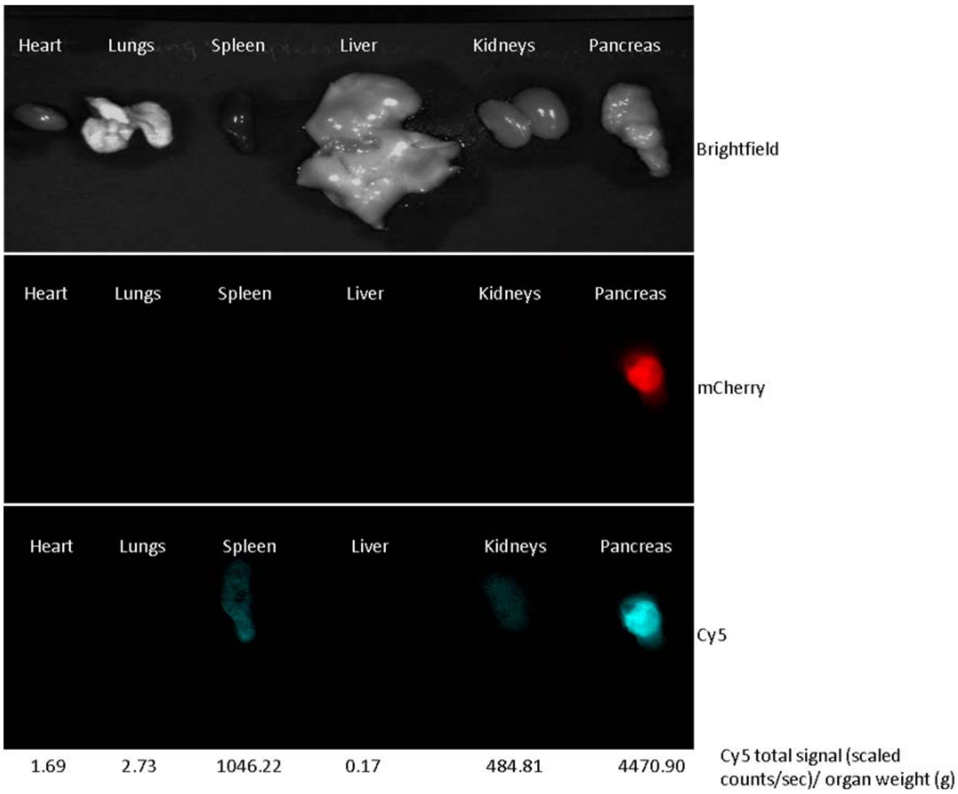

Supplementary Figure 9. Cy5-labeled siRNA delivered by APA accumulated in orthotopic PDAC tumor. Twenty-four hours following intravenous injection of APA poyplexes containing Cy5-labeled siRNA (0.5 mg kg^{-1} siRNA dose) into tumor-bearing mice, tumors and organs were resected and subjected to non-invasive fluorescent imaging (CRI Maestro). Brightfield and fluorescent (Cy5 and mCherry) images of all organs together from a representative mouse treated with the polyplex are presented. Cy5 fluorescent total signals (scaled counts sec^{-1}) were measured from each organ alone and divided by the organ weight (g).

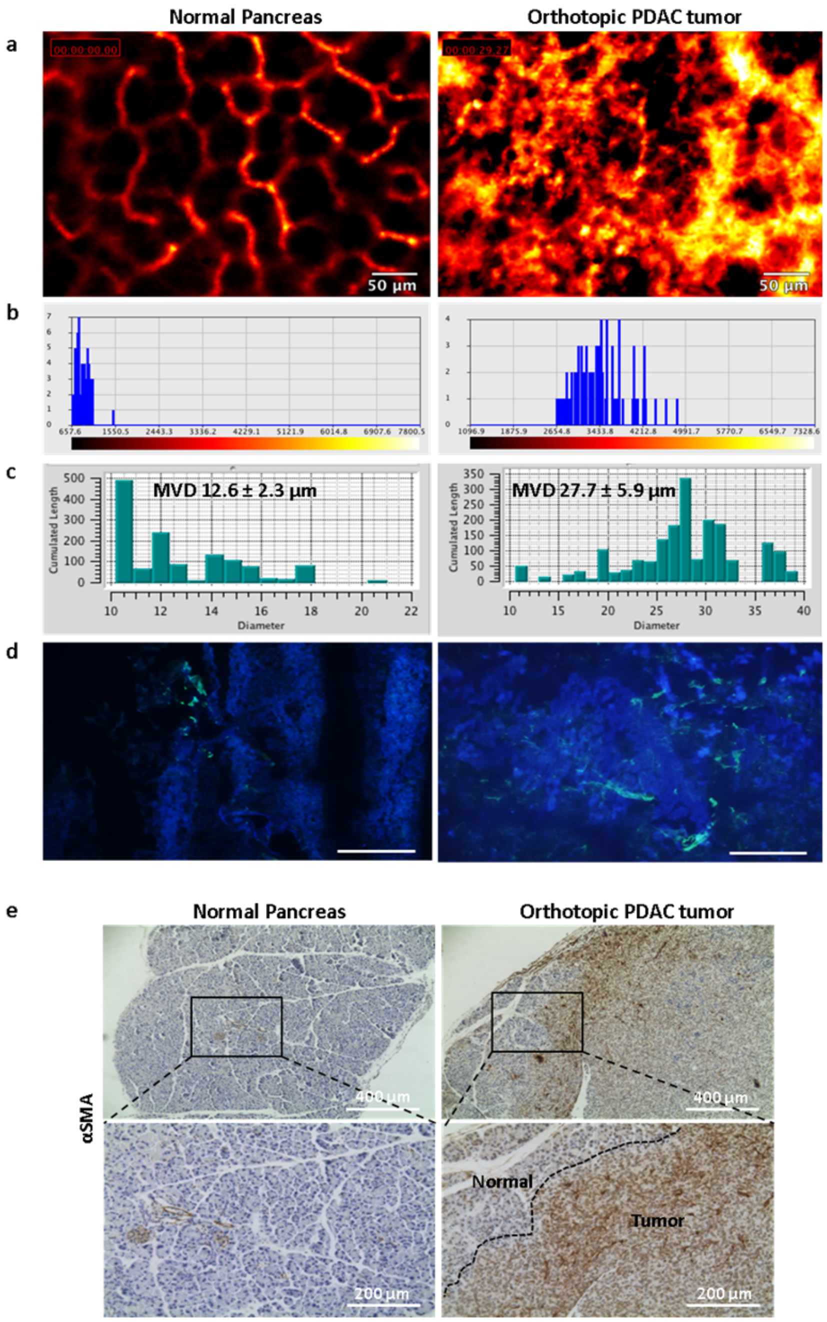

Supplementary Figure 10. Vasculature and stroma morphologies of PDAC xenograft and normal pancreas. a. Following i.v. administration of 70 KDa FITC-labeled dextran to orthotopic tumor-bearing mice, vasculature was monitored by fiber confocal microscopy imaging (CellVizio, Mauna Kea Technologies). Tumor vasculature consists of leaky enlarged blood vessels compared to normal pancreas vasculature. **b**. Fluorescent signal intensity in areas adjacent to blood vessels is presented in a red to white scale (dark red = low signal intensity, white = high signal intensity). PDAC tumor shows higher fluorescent signal outside its blood vessels compared to normal pancreas. **c**. Mean Vessel Diameter (MVD) of blood vessels within PDAC tumor (27.7 µm) is higher compared to the MVD of normal vessels (12.6 µm). **d**. Blood vessels staining of OCT sections using anti-CD31 antibody (in green). Nuclei were stained with DAPI (in blue). Scale bar, 10 µm. **e**. α-SMA immunostaining of FFPE sections showing cancer-associated activated fibroblasts in PDAC tumor.

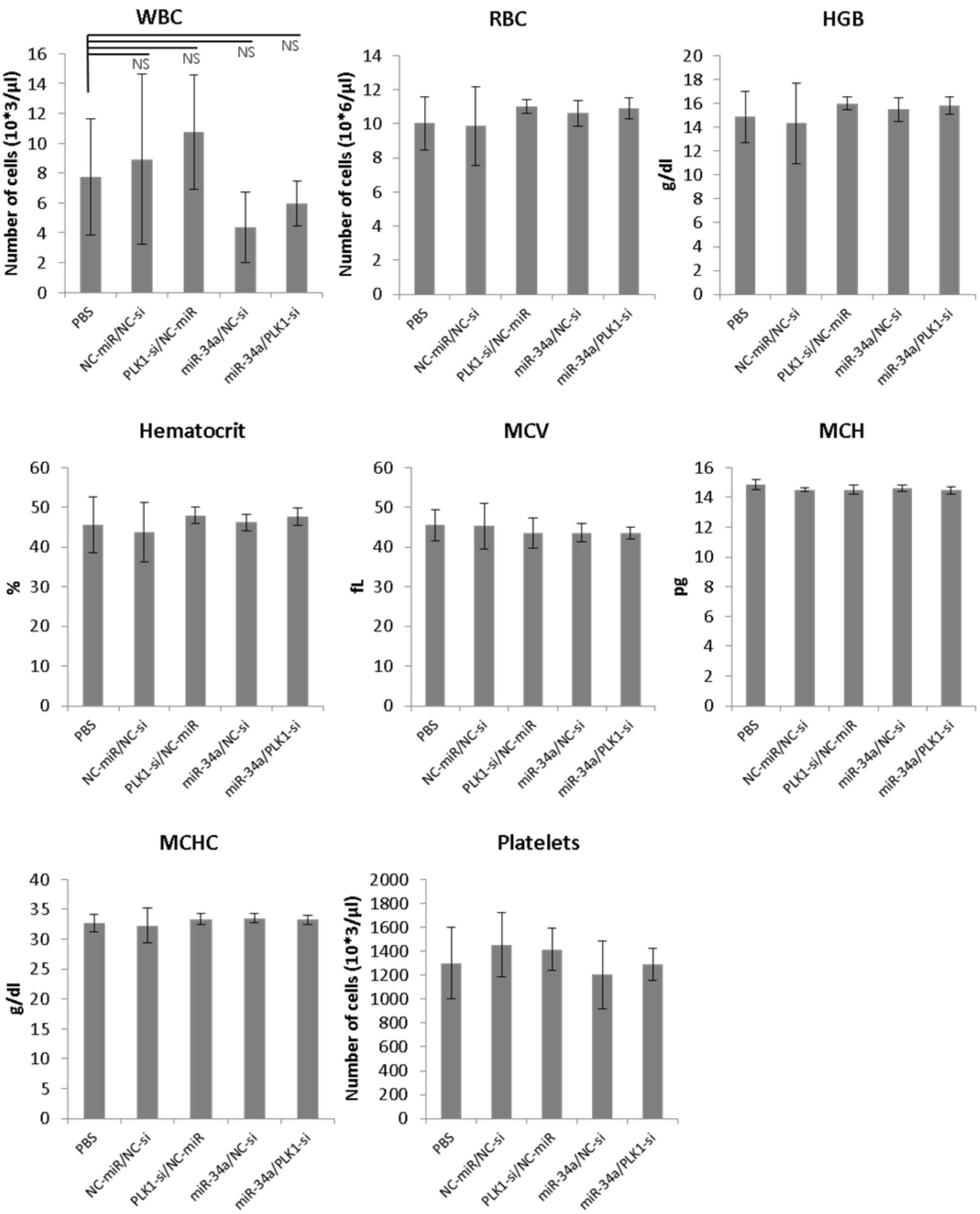

Supplementary Figure 11. Treatment with APA-miRNA-siRNA combination did not induce systemic toxicity. During the *in vivo* efficacy experiment, blood was withdrawn from pancreatic tumor-bearing mice 40 days following tumor inoculation and was analyzed for blood count. WBC, white blood cells; RBC, red blood cells; HGB, hemoglobin; MCV, mean corpuscle volume; MCH, mean corpuscular hemoglobin; MCHC, mean corpuscular hemoglobin concentration. Data represent mean ± SD. (student's *t*-test, NS; Not significant for $P > 0.05$).

	PBS	APA nanocarrier				Reference values
		miR-34a/ PLK1-si	miR-34a/ NC-si	PLK1-si/ NC-miR	NC-miR/ NC-si	
Calcium (mg/dl)	10.52	10.12	10.99	10.54	11.02	9.34-11.55
Phosphate (mg/dl)	6.2	7.7	10	9	9.4	6.93-13.22
Potassium (mmol/L)	6.6	7.6	8.2	7.8	8.7	6.69-10.17
Glucose (mg/dl)	140	140	128	111	123	69.5-158.96
Urea (mg/dl)	41.6	51.5	50.4	45	52.5	15.81-69.41
Cholesterol (mg/dl)	150	126	165	116	153	65.26-170.32
Total protein (g/dl)	6.11	6.04	6.73	6	6.38	5.23-6.95
Albumin (g/dl)	3.7	3.4	4	3.4	3.7	3.27-4.83
Globulin (g/dl)	2.41	2.64	2.73	2.6	2.68	1.4-2.83
Total Bilirubin (mg/dl)	0.07	0.05	0.08	0.06	0.07	0.06-0.3
Alkaline phosphatase (IU/L)	68	67	94	49	68	45.57-214.93
SGOT (IU/L)	86	120	114	93	120	11.68-512.11
SGPT (IU/L)	25	31	35	22	31	0-251

Supplementary Table 3. Blood biochemistry of tumor-bearing mice from the efficacy experiment treated with APA-miRNA-siRNA polyplexes or PBS at day 40 from tumor inoculation.

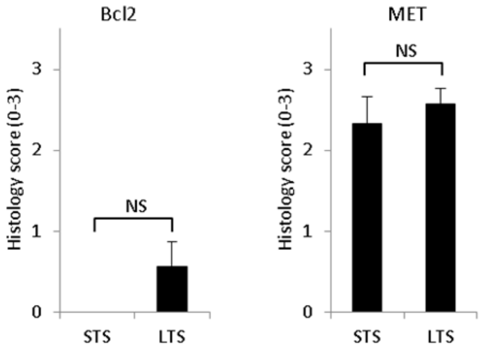

Supplementary Figure 12. Bcl2 and MET immunostaining quantification in FFPE specimens of STS and LTS PDAC patients. Quantification of miR-34a target genes, Bcl2 and MET immunostaining in FFPE samples, based on histology scores (0-3: 0- none, 1- weak, 2- moderate, 3- high) showed no significant difference in their levels between LTS and STS. LTS; Long-term survival, STS; Short-term survival. Data represent mean ± SEM. (Student's *t*-test, NS; Not significant).

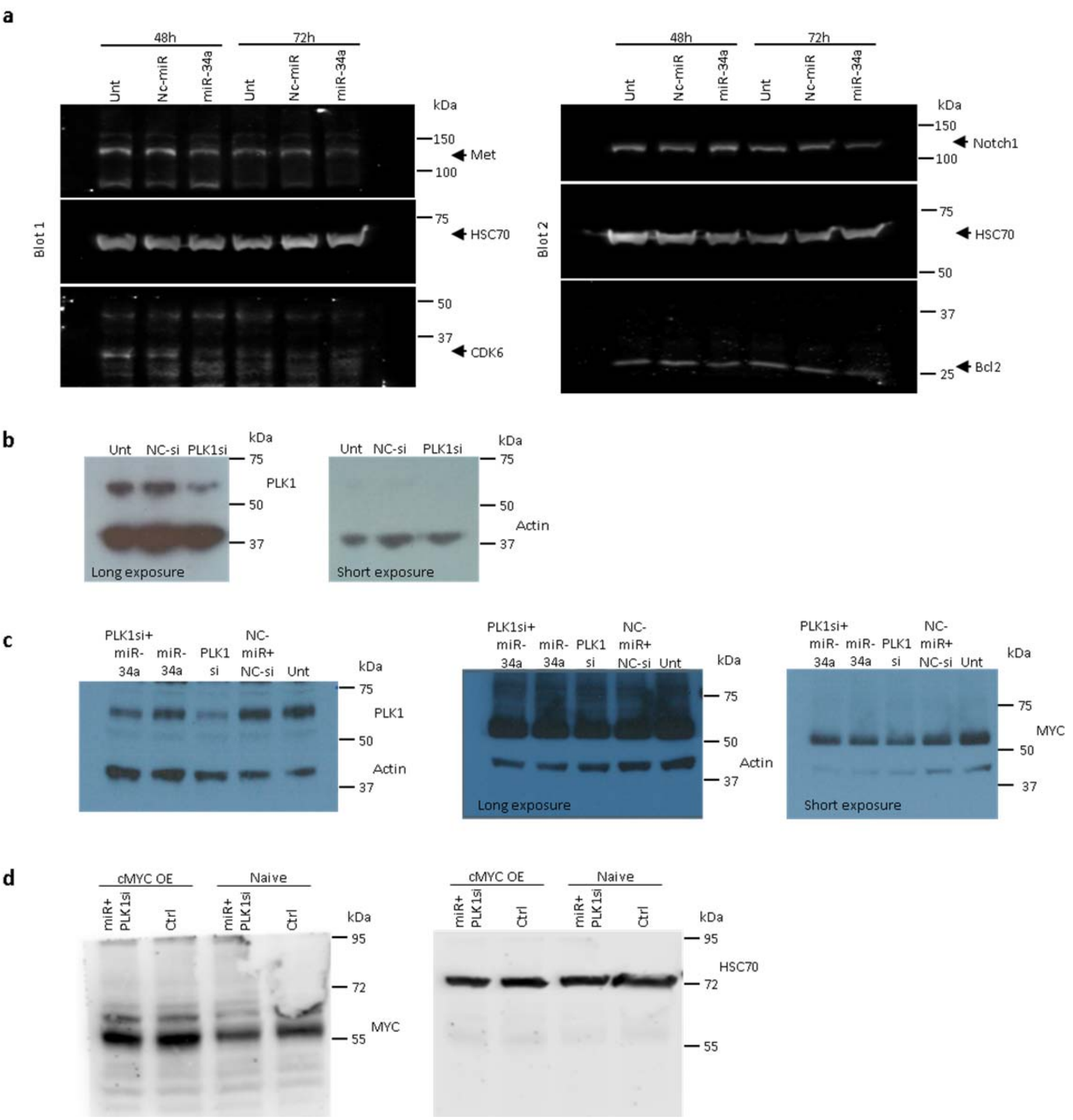

Supplementary Figure 13. Uncropped scans of blots from Fig. 4b (**a**), Fig. 4d (**b**), Fig. 8b (**c**) and Fig. 8f (**d**).
