# Supplementary material for: Amphiphilic nanocarrier-induced modulation of PLK1 and miR-34a leads to improved therapeutic response in pancreatic cancer
Source: Nat Commun. 2018 Jan 2;9:16. doi: 10.1038/s41467-017-02283-9 (PMC5750234; doi:10.1038/s41467-017-02283-9)
Supplement: Supplementary file 3 — Description of Additional Supplementary Files [file 41467_2017_2283_MOESM3_ESM.pdf]

**Description of Additional Supplementary Files**

File Name: Supplementary Movie 1
Description: Nano-polyplexes internalize into PDAC cells via endo-lysosomal pathway.

File Name: Supplementary Movie 2
Description: MiaPaCa2 cell Migration 48 h following incubation with combination treatment.
